# Sleep Disorders in Children with Autism Spectrum Disorder: Developmental Impact and Intervention Strategies

**DOI:** 10.3390/brainsci15090983

**Published:** 2025-09-13

**Authors:** Maria Ludovica Albertini, Giulia Spoto, Graziana Ceraolo, Maria Flavia Fichera, Carla Consoli, Antonio Gennaro Nicotera, Gabriella Di Rosa

**Affiliations:** 1 Unit of Child Neurology and Psychiatry, Department of Human Pathology of the Adult and Developmental Age “Gaetano Barresi”, University of Messina, 98125 Messina, Italy; marialudovica.albertini@gmail.com (M.L.A.); graziana.c23@hotmail.it (G.C.); maria.flavia.fichera@gmail.com (M.F.F.); carlaconsoli@hotmail.com (C.C.); 2 Unit of Child Neurology and Psychiatry, Department of Biomedical Sciences, Dental Sciences & Morphofunctional Imaging, University of Messina, 98125 Messina, Italy; giulia.spoto27@gmail.com (G.S.);; 3 Unit of Child Neurology and Psychiatry, Maternal-Infantile Department, University of Messina, 98125 Messina, Italy

**Keywords:** autism, circadian rhythms, development, EEG, melatonin, sensory processing, sleep disorders

## Abstract

Sleep disorders are highly prevalent in children with Autism Spectrum Disorder (ASD), profoundly impacting their neurodevelopment and daily functioning. Alterations in sleep architecture and regulatory mechanisms contribute to difficulties with sleep onset, maintenance, and overall sleep quality. Sensory processing differences, commonly observed in ASD, may further exacerbate these disturbances by affecting arousal regulation and environmental responsiveness during sleep. Given the fundamental role of sleep in brain maturation, its disruption negatively impacts synaptic plasticity and neurological development, particularly during critical periods. These sleep-related alterations can influence cognitive and behavioral outcomes and may serve as early indicators of ASD, highlighting their potential value in early diagnosis and intervention. Understanding the neurobiological mechanisms linking sleep and ASD is essential for developing targeted therapeutic strategies. Ongoing research increasingly focuses on pharmacological, nutraceutical, and behavioral interventions aimed at mitigating sleep disorders and their cascading effects on neurodevelopment. Optimizing these therapeutic approaches through a multidisciplinary lens is crucial for enhancing clinical outcomes and improving overall quality of life in children with ASD.

## 1. Introduction

Autism spectrum disorder (ASD) refers to a range of early-onset deficits in social communication and repetitive sensory–motor behaviors, arising from a multifactorial etiology involving genetic predisposition, epigenetic mechanisms, and early environmental influences [1,2,3,4]. It is one of the most common neurodevelopmental disorders (NDDs), with a prevalence of up to 1 in 68 children and several factors contributing to this rise (e.g., increased awareness and changes in diagnostic criteria) [5,6].

ASD is frequently associated with co-occurring medical and psychiatric conditions such as intellectual developmental disorder (IDD), attention deficit/hyperactivity disorder (ADHD), anxiety, epilepsy, sleep and gastrointestinal disorders. Although not part of the diagnostic criteria, these comorbidities affect up to 80% of individuals, impacting quality of life and requiring tailored interventions [1,7]. Research shows that up to 86% of individuals with ASD experience at least one sleep problem daily [8,9]. Such sleep disorders tend to negatively affect the daily functioning, elevating their stress levels and placing an additional burden on caregivers [10]. In fact, sleep disruption can have potentially serious consequences, both behavioral and cognitive [11]. Studies showed that sleep deprivation worsens core ASD symptoms, including repetitive behaviors and impairments in social communication. It also intensifies internalizing behaviors such as anxiety, withdrawal, and depression, as well as externalizing ones like aggression, tantrums, and inattention [12,13,14,15,16,17,18]. Reduced total sleep time (TST) has been associated with higher rates of stereotypies as well as higher overall autism severity scores and social skills deficits [19]. The literature highlights that stereotypies can disrupt sleep by competing with behaviors that promote sleep onset and with the state of behavioral quietude necessary for falling asleep [20,21]. Evidence is limited, and further studies are needed to clarify the causal role of different stereotypies and their potential functions in emotional regulation or self-soothing [22]. In addition, individuals with ASD often show a marked preference for routines and rituals, particularly those related to bedtime. Disruption or absence of a consistent bedtime routine can trigger significant distress and maladaptive behaviors, ultimately delaying sleep onset. Engagement in time-consuming rituals or intrusive repetitive thoughts may further lead to cognitive over-activation and emotional hyperarousal, hindering the ability to fall asleep [23]. Thus, there is a bidirectional relationship between sleep and ASD-related characteristics, as behavioral problems could exacerbate sleep difficulties and vice versa [24]. Moreover, sensory abnormalities have also been identified as related to sleep problems in children with ASD. Specifically, a strong correlation has been found between sensory avoidance behaviors and sleep problems [16,25,26].

From a cognitive standpoint, sleep disorders in the general population have been linked to impairments in academic and occupational performance, as well as in neuropsychological functions such as executive functioning [27]. As far as ASD is concerned, the evidence is inconsistent: some studies state that sleep disturbances are significantly associated with poor school performance and cognitive and executive functions in individuals with ASD; others, however, do not indicate any relationship with executive functions and claim that sleep, although interrupted, still effectively stabilizes memory in children with ASD, albeit to a lesser degree than in controls [28,29,30]. These disorders are often underestimated and therefore undertreated in children with ASD [31].

Given the high prevalence and significant impact of sleep disturbances in children with ASD, a deeper understanding of the underlying pathophysiological mechanisms is essential to inform effective therapeutic strategies. This review aims to explore the neurobiological and regulatory mechanisms contributing to sleep disorders in ASD, with a particular focus on how these alterations affect arousal, sensory processing, and circadian rhythms. Furthermore, it will examine current pharmacological and non-pharmacological treatment approaches with the goal of outlining evidence-based strategies to improve sleep quality and, consequently, the overall well-being and developmental trajectories of children with ASD.

## 2. Neurobiology of Sleep in Typical and Atypical Development

Sleep is essential for human life and involves physiological and behavioral processes; it is recognized as a state involving intense brain activity [32,33]. Functionally, it consists of a reduction in responsiveness to weak stimuli and rapid reversibility in response to strong stimuli [34]. Nevertheless, it is not considered merely a resting state but a highly active neurophysiological process that contributes to the efficient formation of cortical circuits [35,36].

In adult sleep, two main stages are conventionally classified using electroencephalographic (EEG) recordings: Non-Rapid Eye Movement (NREM) sleep and Rapid Eye Movement (REM) sleep. The former is characterized by slow-frequency, high-amplitude EEG activity, sleep spindles (bursts of 10–16 Hz activities lasting 0.5–2 s), and reduced muscle tone on electromyogram (EMG); the latter is defined by low-amplitude EEG patterns (especially in the low frequencies) and a suppression of muscle tone on the EMG [37].

NREM sleep is generated by two populations of gamma-aminobutyric acid (GABA)ergic neurons in the preoptic region of the hypothalamus and adjacent basal forebrain. Neurons in the median preoptic area are thought to mediate sleepiness and initiate the transition from wakefulness to NREM sleep, while ventrolateral preoptic neurons are considered crucial for sleep maintenance and regulating REM sleep homeostasis [37,38]. REM-on cells, which are maximally active during REM sleep, utilize neurotransmitters such as GABA, acetylcholine, glutamate, and glycine, whereas REM-off cells, which are minimally active during REM, rely on norepinephrine, epinephrine, serotonin, histamine, and GABA; these two populations are primarily located in the pons, adjacent midbrain regions, and the hypothalamus [13,37,39]. Additionally, melatonin is also involved in the promotion of sleep: it is a hormone synthesized by the pineal gland and has soporific, anxiolytic, and chronobiotic effects [39,40]. Abnormal profiles in melatonin levels have been proposed as possible factors implicated in the sleep disorders of individuals with ASD [41,42]. Furthermore, increased serotonin levels in the blood of children with ASD and in their unaffected relatives have been described [43]. The disruption of sleep–wake cycle networks and depletion of sleep-related brain neurotransmitters have been suggested as main mechanisms underlying the sleep disorders in individuals with ASD [39,40].

The most frequently sleep disturbances encountered in children with ASD include bedtime resistance, delayed sleep onset, middle of the night awakenings or frequent nocturnal awakenings, sleep-related anxiety, daytime sleepiness, parasomnias, and circadian sleep–wake rhythm disorders (CSWRDs) [44,45]. These difficulties are characterized by increased sleep onset latency (SoL), reduced TST, increased wake after sleep onset (WASO), and poor sleep efficiency (SE) [46].

Moreover, sleep-related movement disorders are also common in the pediatric population. They include the restless legs syndrome (RLS), periodic limb movement disorder (PLMD), and a newly described sleep disorder called “restless sleep disorder” (RSD) and characterized by excessive nocturnal motor activity during sleep, such as large body movements or frequent repositioning, despite the absence of sleep onset delay or frequent awakenings [47]. Children with RSD commonly exhibit daytime symptoms, particularly excessive sleepiness, cognitive difficulties at school, irritability, or hyperactivity [48].

Previous studies hypothesized that sleep difficulties may be associated with sensory abnormalities in both ASD and typically developing children [49,50,51,52]. Hollway and colleagues found an inverse relation between the Underresponsive/Sensory Seeking subscale of the Short Sensory Profile (SSP) and the Children’s Sleep Habits Questionnaire (CSHQ), suggesting that children who are underresponsive may miss the environmental cues that entrain the sleep/wake cycle. In the same study, lower scores on the SSP Taste/Smell Sensitivities subscale were associated with increased sleep anxiety, which interfered with bedtime routines and contributed to sleep onset delays [49]. Similarly, Mazourek and Petroski proved that in children with ASD aged 2–5 years sensory over-responsiveness (evaluated with SSP scores) was associated with sleep problems such as sleep-onset delay, sleep duration, and night awakenings; in older children (6–18 years) sensory over-responsiveness correlated with bedtime resistance, sleep-onset delay, sleep duration, and sleep anxiety [50]. Finally, Tzischinsky and colleagues explored the relation between sleep disturbances and hypo- and hypersensitivity scores for each of five sensory domains. The authors found that touch hypersensitivity displayed the strongest relationship with sleep disturbances, suggesting it may interfere with both sleep onset and maintenance in children with ASD [51]. Anxiety represents a crucial factor affecting sleep quality in individuals with ASD. Elevated anxiety levels can increase physiological arousal and hinder the relaxation processes necessary for both sleep initiation and maintenance. This condition has been repeatedly associated with difficulties in falling asleep and frequent night awakenings, supporting the hypothesis of a complex bidirectional interaction between sensory processing, emotional regulation, and sleep quality in this population [49,50].

## 3. Neurodevelopmental Role of Sleep in Infancy

The first recognizable signs of autism typically emerge in the second half of the first postnatal year, with early impairments in social communication and interaction gradually becoming more apparent over the following years, but the neurodevelopmental alterations underlying ASD begin much earlier [53,54,55]. In this context, anatomical abnormalities such as an overall increase in cerebral cortical volume during the first and second years of postnatal life have been demonstrated in infant siblings subsequently diagnosed with autism. Particularly, these individuals exhibited accelerated growth of the cortical surface area from age 6–12 months in comparison with infant siblings who were not later diagnosed with ASD [56].

These early alterations in brain development unfold during a period of rapid neural plasticity and dynamic change [57]. Synaptic plasticity describes the activity-dependent ability of synapses to undergo changes in numbers, through genesis or pruning, or in functional strength [58,59]. It can occur via different mechanisms throughout the lifespan, involving both endogenous and exogenous elements such as neurotransmitters, growth factors, and environmental stimuli [58,60,61,62]. Critical periods are times during development characterized by an extreme form of neuronal plasticity, and brain maturation relies not merely on chronological age but also on experience and an enriched environment [63]. Abnormalities in cortical and brain growth early in life have been implicated in how experience shapes the brain, making these deficits in plasticity important contributors to the ASD phenotype [61,64].

Brain development begins during the early stages of pregnancy and continues into adulthood, with the first two years being the period of most dramatic changes [57,65,66,67]. During this period, infants spend most of their time asleep, with sleep playing a fundamental role in shaping brain architecture [36,68,69]. Sleep architecture undergoes rapid and dynamic changes during early development, with the most prominent modifications occurring in the very first stages of life, reflecting the accelerated processes of brain growth and maturation [69]. Indeed, in the developing brain, sleep is not yet organized into distinct patterns that clearly differentiate it from wakefulness. In preterm infants around 24 weeks post conceptional age, the EEG is marked by discontinuous activity (called “trace discontinue”), consisting of bursts of high-amplitude undifferentiated waves interleaved with periods of silence [70]. During the third trimester of gestation, it gradually evolves into a more continuous, faster, and more synchronous pattern of lower amplitude, within which three behavioral states can be distinguished: active wakefulness, active sleep with REM, and quiet sleep, which will subsequently mature into NREM sleep [69]. In this context, around 28 weeks of postconceptional age, a distinctive pattern of activity emerges during active sleep, characterized by short 5–25 Hz bursts superimposed on a slower 0.5–2 Hz background. These events, known as delta brushes or spindle bursts, represent a hallmark of immature cortical activity [71]. These activities are linked to peripheral inputs and are localized to the corresponding somatosensory cortex, playing an important developmental role [70]. Moreover, this supports the role of spindle bursts in promoting neuronal plasticity; indeed, as early as 1966, Roffwarg and colleagues proposed that the high levels of active or REM sleep observed in early life may provide endogenous stimulation to the developing cortex at a time when external sensory input is limited [68]. The reduction in spindle burst activity with increasing age may contribute to the closure of pre-critical periods of cortical plasticity, marking the transition to the critical period during which sensory inputs begin to refine and shape existing neural circuits [70]. Similarly, adult sleep spindles represent the rhythmic network activity of the brain and have been implicated in synaptic plasticity, especially in learning and memory functions [71,72].

Another spontaneous activity occurring during NREM sleep is the slow-wave activity (SWA), a 0.5–4 Hz oscillation that synchronizes sensory, motor, and association cortices. It has been suggested that this activity may be associated with learning and memory formation and consolidation, playing an important role in synaptic remodeling [70]. In particular, the synaptic homeostasis theory states that learning-related plasticity depends on synaptic potentiation and formation but must be balanced by equivalent reductions in synaptic strength to avoid excessive excitation. Those mechanisms do not occur at the same time but rather unfold sequentially; according to the synaptic downscaling theory of sleep, synaptic potentiation occurs predominantly during wakefulness and is then selectively downscaled during sleep [73]. In this light, SWA facilitates the learning-related changes occurring in the sensory circuits [70].

An excitatory/inhibitory imbalance has been previously hypothesized as one of the potential contributing factors in ASD, suggesting that a disproportionate level of excitation (glutamatergic/GABAergic signaling), mediated by local circuit plasticity mechanisms, could lead to noisy and unstable cortical networks, thereby affecting language and social behavior, as well as perception, memory, cognition, and motor control [74]. Therefore, the relationship between sleep and autism is likely bidirectional and tightly interlinked. Sleep disturbances may contribute to atypical behaviors in ASD by disrupting the excitatory/inhibitory balance, essential for sensory processing and circuit development; conversely, core features of ASD, including excitatory/inhibitory imbalance and sensory hypersensitivity, may in turn impair sleep regulation, suggesting a shared neurobiological mechanism linking sleep and ASD [75].

A critical role in regulating the memory function has been attributed to the hippocampus, which, during sleep, exhibits specific electrophysiological patterns known as sharp wave ripples (SWRs) [76]. These events are formed by the sharp waves, which are large amplitude negative polarity deflections (40–100 ms), and the short-lived fast oscillatory activity superimposed onto sharp waves, known as “ripples” (110–200 Hz) [77]. SWRs primarily occur during NREM sleep and are thought to contribute to memory consolidation processes [76,77]. Studies on ASD mouse models proved alterations in SWRs and particularly decreased power of ripples [78] and slower activity emerging from the dorsal hippocampus [79], underlying their relationship with cognitive functions. Therefore, their disruption may reflect aberrant brain network organization and altered synaptic plasticity, as observed in ASD.

## 4. Sleep as Prognostic Marker of Neurodevelopmental Disorders

The quality and quantity of sleep, especially during the first year of life, are correlated with mental development. Experimental studies show that deprivation of REM or NREM sleep in young animals leads to reduced brain size, impaired learning, and long-term behavioral alterations [35]. Furthermore, sleep is essential for emotional processing, stress management, and overall daily functioning [11]. Disrupted sleep in early life has been linked to reduced social competence, delays in cognitive development, and attention difficulties in young children [80,81,82,83,84,85]. This may be due to overlapping brain structures involved in both sleep and attention, such as the ascending reticular arousal system, which connects to the hypothalamus and thalamus [86]. Particularly, the suprachiasmatic nucleus of the anterior hypothalamus, dorsomedial hypothalamic nuclei, and locus coeruleus constitute the circadian timing system, which is essential for controlling the sleep–wake cycle with a rhythmicity and oscillations close to 24 h [87]. These structures are involved in arousal modulation, regulating alertness, vigilance, attention, learning, and memory [86].

As a result, early sleep disturbances in infants with NDDs could contribute to attention deficits and later challenges in cognition and social skills. For instance, Elkhatib Smidt and colleagues explored how ASD traits and executive functions relate to sleep–wake patterns using actimetry in individuals with ASD and their family members: results showed that greater autism traits and executive dysfunction are linked to disrupted circadian rhythms in adults and to a lesser extent in children [88]. Begum-Ali and colleagues also focused on the close link between sleep and neurodevelopmental outcomes, investigating the association between early sleep patterns, early visual attention, and later emerging ASD and/or ADHD symptomatology, as well as with cognitive and social adaptive skills. The authors confirmed that poor night sleep in infancy is related to ASD traits and reduced socialization, with stronger effects from 14 months onward; they also proved that early sleep may influence visual attention development [89].

In terms of implications for long-term cognitive and executive impairments, Mazurek and colleagues found that sleep disturbance is a longitudinal predictor of the development of hyperactivity and attention problems in children with ASD [16]. It has been suggested that the relation between ASD and sleep difficulties might be due to atypical developmental trajectories of hippocampus, in light of its crucial role in the mediation of cognitive processes. In fact, in their study on 432 infants at high-risk or low-risk for ASD, MacDuffie and colleagues found that infants that developed ASD had significantly higher sleep onset problems between 6 and 12 months. Moreover, difficulties with sleep onset during infancy were associated with weaker social communication skills by 24 months and were related to hippocampal volume trajectory from 6 months to 24 months only for the high-risk ASD group [90].

Regarding communication skills, Hollway and colleagues retrospectively evaluated risk markers and correlations of sleep disturbances in 1583 children with ASD and found an inverse association between the Communication Domain of the Vineland Adaptive Behavior Scale and Sleep Duration scores of the CSHQ The authors highlighted the close link between sleep and communication deficits (a core symptom of ASD), suggesting that sleep problems may hinder the acquisition of communication skills during the day. Conversely, lower communication scores could predict sleep dysfunction, while ASD symptom severity appeared to predict sleep outcomes [49]. Sleep deprivation has also been associated with cognitive and socio-emotional impairments, as well as poorer performance on verbal fluency tasks [91].

Some authors found that early sleep disorders not only exacerbate ASD core symptoms but also increase maladaptive behaviors especially worsening emotional regulation [11,30,92]. Although the link between sleep disturbances and behavioral problems in children with ASD is well established, few studies have explored sleep issues over time and their specific impact on emotional regulation in very young children with ASD or other NDDs. Children with ASD are more prone to emotional dysregulation, which represents a key factor in functional impairment [93,94,95]. In a cross-sectional and longitudinal prospective study conducted on 136 very young children with NDDs (88 with ASD and 48 with NDDs with or without ASD), Favole and colleagues showed that after a mean period of 17.2 months sleep disturbances correlated with more severe emotional disregulation, regardless of age, cognitive development, and ASD symptom severity [96]. Moreover, a significant predictive relationship between prior sleep disruption and daytime behavior has also been proved by Cohen and colleagues. In their study on 67 autistic children and adolescents, they demonstrated that mean SE and variability in SE and sleep onset were associated with a higher probability of challenging behavior in the following days; on the contrary, mean sleep onset time, mean TST, and variability in sleep offset time were negatively correlated with behavioral problems [11].

Recent studies confirmed a bidirectional relationship between sleep disorders and emotional dysregulation, where poor sleep impairs emotional control and emotional dysregulation in turn worsens sleep [96,97,98,99]. This notion is also supported by both caregiver-reported and actigraphy-based sleep data, which show that disrupted or inconsistent sleep patterns correlate with increased anxiety and externalizing behaviors in ASD [100,101,102,103]. Particularly, in children/adolescents with ASD, arousal disorders and excessive somnolence are related to greater thinking and behavioral problems [104].

Collectively, this evidence suggests that early sleep disturbances (especially short TST, frequent night awakenings, and settling issues) are among the earliest predictors of ASD, with sleep disruption from infancy linked to later ASD traits, cognitive, and social difficulties [84,89,90,105].

## 5. Heterogeneity of Sleep Disturbances in ASD

Sleep problems are highly prevalent in ASD, though they do not present uniformly across the spectrum. Differences in age, cognitive functioning, and medical comorbidities may substantially influence both sleep architecture and treatment response, even if results in this regard remain unclear. For example, Favole and colleagues found no association between sleep disturbances and the presence of IDD or global developmental disorder, consistent with findings that “poor sleepers” with ASD do not differ in age or cognitive level from “good sleepers” [96]. Some authors reported a positive association between IDD and sleep disorders [106], while others found no link to intelligence quotient (IQ) [15]. Among the formers, Cohen and colleagues showed that sleep disruption predicts daytime challenging behaviors, particularly in individuals with low-functioning ASD (e.g., individuals with severe intellectual and social-communication impairment), where poor SE correlates with higher rates of aggression and self-injury [11].

Masi and colleagues conducted a study to investigate the relationship between sociodemographic variables, such as age and sex, and total CSHQ scores . They highlighted some differences across the age range, with greater levels of bedtime resistance in preschool children while higher levels of daytime sleepiness, greater sleep onset delay and reduced sleep duration were observed in adolescents [44]. Sleep issues in adolescents may worsen due to puberty-related circadian shifts, leading to later sleep and wake times. Combined with early school start times, this often results in insomnia symptoms and daytime fatigue in about half of adolescents [46]. These circadian disruptions can persist into early adulthood, frequently presenting as delayed sleep–wake phase disorder and insomnia. In fact, adults with ASD, particularly those with IDD, tend to show higher rates of sleep issues—such as longer SoL, more WASO, and lower SE—compared to non-autistic adults [46]. Elkhatib and colleagues showed that in adolescents and adults with ASD, poor sleep as measured by polysomnography was associated with cognitive traits, such as declarative memory, selective attention, sensory-motor procedural memory, and cognitive procedural memory; conversely, they reported that in a study of children 7–11 years old, no association was found between sleep–wake patterns and executive functions [88].

Regarding sex differences, it has been seen that autistic females experienced more severe sleep problems than males, including higher bedtime resistance, shorter sleep duration, greater sleep anxiety, and increased daytime sleepiness [44].

Considering the large field of medical comorbidities of ASD, many researchers examined the link between these conditions and sleep disorders in ASD patients. For instance, medical comorbidities such as epilepsy, ADHD, or gastrointestinal disorders may complicate both diagnosis and management of sleep disturbances in ASD. Indeed, epilepsy and abnormal EEG activity can directly disrupt sleep continuity, while ADHD symptoms often worsen SoL and night awakenings [7].

Hollway and colleagues identified anxiety as the strongest cross-sectional predictor of sleep disturbance, along with elevated IQ, greater ASD severity, sensory sensitivities, and gastrointestinal issues also showed significant associations [106].

Hyperactivity, combined or not with inattention, has been seen to be linked to reduced TST, more WASO, and parasomnias, at all ages [46]. Moreover, longitudinal analyses showed that poor sleep predicts later ADHD traits in autistic toddlers but not in older children. At the same time, sleep difficulties may contribute to attention issues and ADHD-mimic behaviors. Finally, evidence showed that behavioral sleep interventions (e.g., sleep hygiene, extinction methods, bedtime fading) not only improve sleep in children/adolescents with ASD but also reduce ADHD symptoms, supporting a behavioral link between ASD, ADHD and sleep problems [46].

## 6. Clinical Approach to Sleep Disturbances in ASD

The treatment of sleep disorders in children with ASD should be considered a therapeutic priority, as it has a profound impact on the quality of life of both patients and their families [107]. Sleep disorders in children with ASD are frequently underrecognized, as clinical attention tends to focus on daytime behavioral issues. A comprehensive assessment should include a detailed sleep history including bedtime, wake time, daytime napping, and night-time awakenings, as well as an evaluation of daytime functioning, including hyperactivity and daytime sleepiness [26]. Clinicians may benefit from using questionnaires that assess multiple dimensions of sleep disturbances, including sleep-related breathing disorders, sleep anxiety, bedtime resistance, and daytime sleepiness [108]. A comprehensive metabolic screening—including the assessment of amino acids, vitamins (particularly vitamin D), iron, and magnesium—may be useful to identify potential deficiencies that could contribute to sleep disturbances and neurodevelopmental dysfunctions in children with ASD [109,110,111,112,113].

Polysomnography remains the gold standard for evaluating sleep in children with ASD, particularly for detecting conditions such as sleep apnea, seizures, parasomnias, and periodic limb movements [24,114,115,116]. However, its use may be limited by factors such as cost, availability, and low tolerance among children. Desensitization protocols can enhance a child’s ability to undergo polysomnography. As a less invasive alternative, actigraphy can be especially useful in documenting sleep–wake patterns, particularly in cases of insomnia or in children with tactile sensitivities or anxiety in unfamiliar environments such as sleep laboratories [26].

In a stepwise approach, initial management typically involves parent education, sleep hygiene, and behavioral interventions aimed at promoting healthy sleep habits. If these non-pharmacological strategies prove ineffective, pharmacological treatments should be considered as adjunctive therapy to ongoing behavioral treatment [25,107,117]. Such treatments may help, at least in part, to alleviate families’ distress and give them something positive to focus on [118].

### 6.1. Non-Pharmacological Interventions for Sleep Disorders in ASD

According to the current guidelines issued by the American Academy of Neurology for the treatment of insomnia and disrupted sleep behaviors in children and adolescents with ASD, a combined approach involving both behavioral and pharmacological strategies is recommended. Once potential contributing factors have been addressed, parental education and behavioral strategies are considered the first-line approach to support the development of healthy sleep habits, although robust evidence remains limited [119]. When implemented and maintained, parent training can be a valuable tool for enhancing parental self-efficacy and the consistent application of behavioral sleep intervention techniques [120]. Malows and colleagues concluded that parent sleep education training (individual or in group) combined with behavioral techniques should be the first approach to improving sleep difficulties in individuals with ASD [121].

Various non-traditional interventions have been explored to promote good sleep habits and improve sleep in children with ASD, particularly those with sensory sensitivities. Weighted blankets and vests provide deep pressure stimulation, which may reduce physiologic arousal and stress, although evidence of efficacy is limited and safety considerations remain important [122]. Massage therapies, including Qigong and Thai massage, may help promote relaxation and reduce bedtime behavioral challenges, although evidence and results are inconsistent [123]. Sound-to-sleep mattresses and regular physical activity have shown potential benefits for sleep quality and duration [124]. Overall, good sleep habits for children with ASD and sensory difficulties include maintaining a low-stimulation environment, consistent bedtime routines, gentle sensory supports, physical activity during the day, and individualized approaches that consider each child’s sensory profile and preferences [107].

For children, several specific behavioral intervention strategies based on learning and behavioral principles have been developed. These strategies are adapted to the type of sleep disorder and aim to promote positive sleep habits, as well as the child’s ability to relax and self-soothe. The main behavioral approaches described in the literature and applied in clinical practice include extinction, scheduled awakenings, bedtime fading, stimulus fading, and chronotherapy. Additional strategies include positive reinforcement, bedtime pass, and cognitive techniques [107,125]. The success of behavioral interventions depends on the involvement of motivated caregivers and trained professionals who apply the strategies consistently. Caregiver engagement is crucial, as these interventions are typically parent-mediated. A summary of the most evidence-based interventions is provided in Table 1.

### 6.2. Oral Non-Prescription Treatments and Nutritional Supplements

When behavioral interventions prove insufficient, oral non-prescription pharmacological options may be considered as adjunctive strategies. Commonly used oral non-prescription treatments and nutritional supplements for sleep problems in children with ASD include tryptophan (Trp), carnosine, iron, magnesium, and vitamin D. These agents are generally well tolerated and may contribute to improvements in sleep onset and maintenance, although robust evidence from randomized controlled trials (RCTs) remains limited [107,125].

#### 6.2.1. Tryptophan/5-Hydroxytryptophan

Trp is an essential plant-derived amino acid that must be obtained through the diet and is required for the in vivo biosynthesis of several compounds, including niacinamide (vitamin B3), serotonin, melatonin, and tryptamine [126]. It was first used in the 1980s for the treatment of sleep disorders and for headache prophylaxis [127].

The mechanisms through which Trp and its metabolite 5-hydroxytriptophan (5-HTP) influence sleep are not yet fully understood. However, it is well established that 5-HTP, obtained from hydroxylation of Trp, increases serotonin levels in the central nervous system, providing a precursor for melatonin synthesis and enhancing the serotonin-mediated regulation of sleep [128]. Once thought to induce slow-wave sleep and later considered wake-promoting, the serotonergic system appears to exert complex effects on sleep that depend on its degree and timing of activation. Serotonin may directly inhibit sleep but subsequently induce a cascade of physiological processes that facilitate sleep, possibly through still unidentified sleep-promoting factors [107].

Several positive effects of Trp on sleep have been reported in adults, including reduced SoL and decreased WASO [127]. It does not produce opioid-like effects nor inhibit desired arousals from sleep, resulting in no or minimal alterations to the physiological architecture of sleep [126].

In the pediatric population, both Trp and 5-HTP have shown efficacy in reducing NREM parasomnias and nocturnal awakenings [129,130].

For instance, in a clinical trial on 165 children and adolescents with primary NREM parasomnias, van Zyl and colleagues demonstrated that 84% of those treated with Trp (dose range: 500–4500 mg/day; mean dose: 2400 mg/day) experienced an improvement in parasomnia symptoms, compared to only 47% in the non-treated group [129]. Similarly, another clinical trial conducted by Bruni and colleagues highlighted that in a cohort of 45 children with sleep terrors, those receiving 5-HTP (2 mg/kg/day at bedtime) showed significantly greater improvement than non-supplemented patients, with sustained effects at six-month follow-up [130].

Trp and 5-HTP are generally safe and well-tolerated, with few and mild side effects. However, due to the risk of serotonin syndrome, they should not be used in combination with certain antidepressants. Adverse effects such as tremor, nausea, and dizziness have been reported, mainly at high doses (70–200 mg/kg/day) or when combined with Selective serotonin reuptake inhibitors (SSRIs) [131]. About 5-HTP, nausea and vomiting may occur at the start of treatment, but these effects are typically mild and transient and can often be avoided by starting with low doses and gradually increasing them if needed [131].

Although experimental studies specifically addressing the effects of Trp/5-HTP on sleep in ASD patients are still lacking, numerous pieces of evidence indicate that dysregulation in Trp metabolism may influence ASD symptomatology [132]. Several studies have documented a significant negative correlation between Trp levels and the severity of ASD symptoms, suggesting that Trp supplementation may help alleviate core behavioral disturbances such as anxiety, irritability, and social impairments [113,133,134]. Reduced dietary Trp intake has been associated with an exacerbation of core ASD traits in both humans and mouse models, which were conversely enhanced by Trp supplementation [135,136,137,138]. Moreover, in a very recent study evaluating the correlation between Trp metabolites and ASD symptoms, higher levels of anthranilate were significantly correlated with greater quality of sleep [132].

Evidence from the literature also demonstrates that children with ASD frequently present with significantly reduced Trp levels, resulting in decreased serotonin availability [134]. Given the critical role of serotonin in regulating mood, behavior, and cognitive function, this alteration may contribute to the affective and behavioral disturbances commonly observed in ASD [113].

Although numerous studies have investigated tryptophan levels [131,132,133,134] and the consequences of acute tryptophan depletion [135,136,137], providing evidence for its role in supporting serotonergic function and behavioral regulation in individuals with ASD, research on tryptophan supplementation in this population remains scarce. Given the encouraging findings reported in pediatric populations with sleep disturbances [129,130], further exploration in ASD could yield important therapeutic implications.

#### 6.2.2. Carnosine

Carnosine is a dipeptide consisting of β-alanine and L-histidine, which acts as an antitoxic and neuroprotective agent in the brain and muscles [139]. Circulating levels of its two precursors have been found to be lower in individuals with ASD compared to controls [140]. Based on these findings, and the high levels of oxidative stress observed in these patients, some authors have proposed that supplementation with 800 mg/day of carnosine may improve communication, social attention, and behavior in children with ASD [141]. However, the evidence regarding the efficacy of carnosine supplementation remains limited and inconsistent.

For instance, Mehrazad-Saber and colleagues conducted a double-blind, randomized, placebo-controlled trial on carnosine supplementation in 43 children with ASD. The results showed significant improvement in sleep duration, parasomnias, and a reduction in daytime sleepiness, without notable side effects [139]. Conversely, Ann Abraham and colleagues conducted a RCT on children with mild to moderate ASD, who were administered carnosine at a dose of 10–15 mg/kg in 2 divided doses. In contrast with the previous study, they observed sleep disturbances including difficulty going to bed and increased hyperactivity, probably due to the twice-daily administration [142]. Despite these initial findings, several methodological issues constrained the studies: in the case of the study by Mehrazad-Saber and colleagues, it should be noted that the small sample size reduced statistical power, as well as participant age heterogeneity and potential bias introduced by limited parental awareness and education regarding autistic behaviors [139]. Similarly, as regards the study by Abraham and colleagues, although the use of the BEARS sleep screening tool, a sleep-specific assessment scale, was a strength, it remained a subjective measure. The study was further limited by its non-double-blind design and a small sample size with a narrow age range, which may have affected the generalizability and introduced bias [142]. On the contrary, the study by Chez and colleagues benefitted from a double-blind design, inclusion of a control group, and the use of validated assessment tools across multiple developmental domains. However, its focus on a narrow age range and the exclusion of certain comorbidities and etiologies may limit the generalizability of the findings, particularly in relation to factors that can influence sleep–wake patterns [141].

Further large-scale, well-controlled trials are warranted to clarify carnosine’s efficacy and define optimal dosing schedules, particularly in relation to sleep disturbances in ASD.

#### 6.2.3. Iron

Iron is an essential nutrient critical for neurological functioning. It is required for the synthesis of hemoglobin and serves as a cofactor for tyrosine hydroxylase, the enzyme that catalyzes the conversion of L-tyrosine into dopamine. In the blood, iron is transported bound to transferrin and stored in monocyte-macrophage cells via ferritin, a protein commonly used as an indicator of peripheral iron stores [143]. Given dopamine’s involvement in sleep regulation, iron plays an indirect role in maintaining the sleep–wake cycle. Its deficiency can impair dopamine synthesis, potentially leading to structural and functional damage in brain areas involved in sleep regulation, such as the hippocampus and basal ganglia [136,144]. Iron deficiency is particularly common in children, especially those with ASD, often due to selective and/or restricted eating habits. Several studies have identified correlations between low iron levels and sleep-related movement disorders, including RLS and PLMD [145].

During infancy, iron deficiency has been negatively associated with sleep organization, leading to shorter TST, increased nocturnal awakenings, greater motor activity during sleep, and difficulties in emotional regulation [146,147]. Additionally, low serum ferritin levels have been linked to increased sleep fragmentation resulting in reduced SE, although a causal relationship has not been established [148]. Dosman and colleagues evaluated the effect of iron supplementation (6 mg/kg/day of elemental iron) on 33 children with ASD and restless sleep. They found a significant improvement in restlessness during sleep, as well as increases in mean ferritin levels, hemoglobin, and mean corpuscular volume [149]. Moreover, in a randomized placebo-controlled trial conducted in 20 children with ASD and ferritin levels at the lower limits of the normal range, Reynolds and colleagues evaluated the effect of ferrous sulfate administered three times daily. Although the study did not show a significant improvement in SoL and WASO measured by actigraphy, a slight significant improvement on the Sleep Clinical Global Impression scale was reported [150]. Among these studies on iron supplementation in pediatric ASD, only the latter employed a rigorous methodology, although the sample size remained limited [150]. Other available studies did not specifically evaluate children with ASD, which limits their direct applicability to this population [146,147].

Based on current evidence, several authors recommend assessing sleep disturbances and monitoring serum ferritin levels in children with NDDs, including ASD, particularly when poor sleep quality is reported. If ferritin levels are <50 ng/mL, supplementation with 1–2 mg/kg/day of elemental iron (up to 6 mg/kg/day of elemental iron) should be administered in 2–3 divided doses [125].

Overall, the evidence in pediatric ASD is limited by small sample sizes, short follow-up, and heterogeneous designs [148,149,150], suggesting that current results should be interpreted with caution and that further large-scale RCTs are needed to establish efficacy and optimal dosing. Nevertheless, assessing iron status should be part of routine evaluation in children with ASD and disrupted sleep, especially when dietary selectivity is present.

#### 6.2.4. Magnesium

Magnesium, a key regulator of neuronal excitability and synaptic transmission, has shown potential in improving sleep onset and overall sleep quality. In adult populations, magnesium is widely used for nocturnal leg cramps and has also been considered for the management of sleep disorders characterized by motor hyperactivity such as RLS and PLMD. However, its effectiveness in these conditions has yielded inconsistent results, with only few studies suggesting that magnesium may reduce symptom severity and improve sleep quality [151,152,153].

In pediatric populations, sleep-related movement disorders are frequently associated with neurodevelopmental conditions and have been linked to both neurotransmitter dysregulation and micronutrient deficiencies [152,154]. Moreover, some authors have found that children with more severe forms of ASD exhibit reduced serum magnesium levels, suggesting a potential role for supplementation even in the absence of overt sleep symptoms [155,156]. Given these associations, magnesium supplementation may represent a cheap and relatively safe therapeutic option for individuals presenting with ASD and sleep-related movement disorders such as RLS and PLMD.

Current evidence on magnesium supplementation for RLS and PLMD remains preliminary, as it is based on studies with small sample sizes, short follow-up periods, and primarily subjective outcome measures [151,152,155,156]. Larger randomized trials with objective sleep assessments are needed to clarify its efficacy. In conclusion, despite limited and somewhat inconsistent evidence, magnesium supplementation appears to be a promising adjunctive strategy for managing sleep-related motor disturbances in children with ASD, particularly in those with documented hypomagnesemia or co-occurring neurodevelopmental comorbidities.

#### 6.2.5. Vitamin D

Vitamin D is a pro-hormone classified among fat-soluble vitamins. It is synthesized endogenously in the skin following exposure to ultraviolet B (UVB) radiation, which converts 7-dehydrocholesterol into pre-vitamin D3 [107]. Vitamin D plays a role in dopamine metabolism and, by influencing Trp hydroxylase activity, may also modulate the conversion of Trp into 5-HTP [125]. However, its potential role in sleep remains unclear and is currently under investigation.

Given its involvement in both dopamine and serotonin pathways, it may be clinically relevant to assess vitamin D levels in children with NDDs who present with insomnia and motor hyperactivity during sleep. In such cases, evaluation of vitamin D status alongside serum ferritin levels may aid in the identification of potentially modifiable contributors to sleep disturbances [125].

### 6.3. Pharmacological Therapy

In more severe cases, when behavioral interventions are not sufficient, pharmacological treatment may represent an effective strategy, given the unique needs of the ASD population [125]. Clinical guidelines for the pharmacological treatment of sleep disorders in individuals with ASD remain scarce, and no specific drugs have been officially approved for this indication [39]. At present, neither the U.S. Food and Drug Administration (FDA) nor the European Medicines Agency have authorized any medication for managing insomnia in pediatric populations, including neurodivergent children. As a result, all available pharmacological options are prescribed off-label [125,157]. Early intervention for insomnia is crucial to prevent its chronicity and to avoid more severe problems in affected individuals, as well as family dysfunction and maternal depression [125,158].

**Figure 1:**
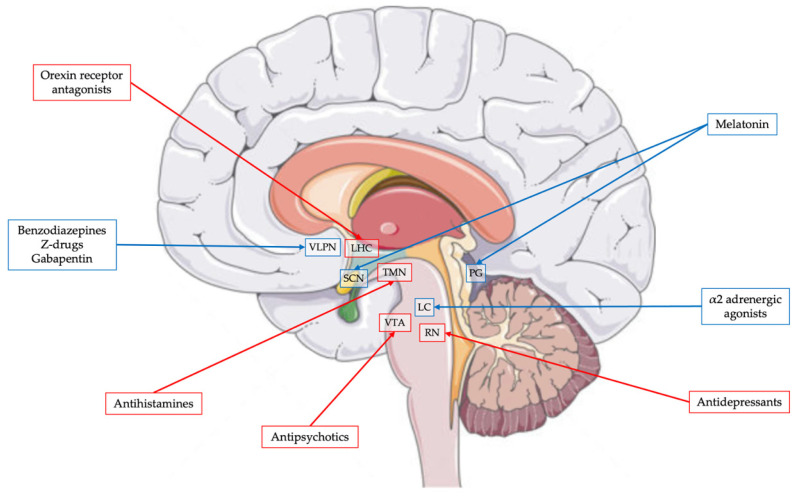
Neuroanatomical structures implicated in the regulation of sleep (blue) and wakefulness (red), serving as principal targets for pharmacological interventions that facilitate sleep through modulation of sleep-promoting and arousal-related areas. Legend: LC: locus coeruleus; LHC: lateral hypothalamus cortex; PG: pineal gland; RN: raphe nucleus; SCN: suprachiasmatic nucleus; TMN: tuberomammillary nucleus; VLPN: ventrolateral preoptic nucleus; VTA: ventral tegmental area.

Both in neurotypical children and those with ASD or, more generally, other NDDs, melatonin is the most prescribed medication, followed by α2-adrenergic agonists, and antihistamines [159,160]. Before initiating pharmacological therapy for sleep disorders, it is essential to consider the patient’s age and clinical history, as well as assess the potential use of other medications for pre-existing conditions, such as anti-seizure medications or antipsychotics. Attention must also be paid to potential drug–drug interactions. Finally, therapeutic goals should be clearly defined, realistic, and measurable, to ensure appropriate monitoring of treatment outcomes [158]. Figure 1 includes the structures implicated in sleep–wake regulations, that represent the main targets for pharmacological interventions. Table 2 summarizes the mechanisms and effects of the most used sleep-promoting drugs, including usual dosages and adverse effects.

#### 6.3.1. Melatonin

Melatonin (N-acetyl-5-methoxytryptamine) is a serotonin-derived neurohormone primarily secreted by the pineal gland. In children and adults, its endogenous production follows a circadian rhythm, beginning in the evening and peaking between 2:00 and 4:00 a.m., and is suppressed by light [107]. Among its various biological functions (e.g., antioxidant properties, anti-inflammatory effects, and roles in the early development of neurons and glial cells), melatonin plays a central role in establishing and regulating circadian rhythms, while also exerting a hypnotic effect through its action on specific melatonin receptors (MTs), namely MT1 and MT2. These receptors are expressed in different brain regions involved in the control of both REM and NREM sleep [126].

Given its well-established chronobiotic and sleep-promoting properties, as well as its perception as a “natural” substance owing to its endogenous origin, melatonin is one of the most prescribed agents for children and adolescents with sleep disturbances. Particularly, melatonin is the most used treatment for insomnia and CSWRDs in individuals with ASD [39]. Current evidence supports melatonin’s efficacy in reducing SoL, advancing the sleep phase, and increasing TST, as shown by subjective tools (scales/questionnaires/diaries) and partially confirmed by actigraphic data. However, its impact on nocturnal awakenings is limited, likely due to its short half-life about 40 min [139,161]. No serious adverse events have been associated with melatonin use. Recent larger studies have confirmed its effectiveness and safety, showing mild side effects, such as fatigue, daytime sleepiness, and headache [107,162].

Depending on the type of sleep disorder, different melatonin formulations may be used, such as controlled release, prolonged release (PedPRM), immediate release, and fast release (FR) [163]. PedPRM is recommended for children who have difficulty maintaining sleep, whereas FR formulations are preferred for children with sleep-onset difficulties [163]. To date, no standardized melatonin dosage has been established. Thus, dosing should be tailored to the type and severity of sleep disorder [162]. In general, dosages range from 1 to 3 mg per night (up to 5 mg in adolescents), with only minimal additional benefit observed at doses exceeding 9 mg per night [45]. In clinical practice, the timing of melatonin administration is a key determinant of its therapeutic efficacy. Phase response curve studies have demonstrated that a 0.5 mg dose of melatonin produces the greatest phase advances when administered approximately 2 to 4 h before the onset of endogenous melatonin secretion (dim light melatonin onset, DLMO), which corresponds to roughly 9 to 11 h before the sleep midpoint [164]. In this case, melatonin acts primarily as a chronobiotic agent for delayed sleep phase disorder. In contrast, the most significant phase delays are observed when melatonin is taken 12–15 h after DLMO or within the first few hours following morning awakening. Consequently, melatonin taken 30–60 min before bedtime facilitates sleep onset, exerting minimal effect on circadian phase shifting. In these cases, melatonin acts mainly as a hypnotic agent rather than a chronobiotic. Notably, the strongest phase-shifting effects occur when endogenous melatonin levels are low, highlighting the need for precise timing in treatment protocols targeting circadian rhythm disorders [164].

In a randomized, double-blind, placebo-controlled trial involving 125 pediatric patients with ASD or Smith-Magenis syndrome, Gringras and colleagues demonstrated that PedPRM mini-tablets (ranging from 2 to 10 mg) significantly improved TST, with a mean increase of 32 min, and reduced SoL by an average of 25 min, ameliorating longest continuous sleep period. Notably, 41% of participants responded to the low-dose of 2 mg of PedPRM and did not require dose escalation [162].

In a multicenter RCT involving 196 children with ASD and prolonged SoL (≥30 min), Hayashi and colleagues reported a significant reduction in SoL in the groups treated with 1 mg of melatonin (−22.0 min) and 4 mg of melatonin (−28.0 min), compared to the placebo group (−5.0 min) [165].

From a practical standpoint, a challenge in melatonin therapy is the loss of efficacy after an initially positive response. This phenomenon may be explained by the slow melatonin metabolism potentially linked to a single nucleotide polymorphism in the CYP1A2 gene [162,166]. Impaired metabolism can lead to accumulation of melatonin during daytime hours, resulting in the disruption of the circadian melatonin rhythm and a subsequent decline in the effectiveness of exogenous melatonin over time. Consequently, there is increasing recognition that low doses, such as 0.5 mg, may be effective for some children, while doses exceeding 6 mg are often associated with diminishing therapeutic benefit [166].

Finally, Ramelteon, a synthetic agonist of the MT1 and MT2 receptors, approved in adults, has shown limited efficacy on nocturnal awakenings in a few clinical cases conducted in children with NDDs [125].

In conclusion, melatonin is the most well-studied and commonly prescribed agent for sleep disturbances in children with ASD, with RCTs and meta-analyses supporting its efficacy and safety profile, showing only mild adverse events [162,163,165]. Notably, the most recent meta-analysis in pediatric ASD populations corroborates these findings, confirming that melatonin is both effective and safe compared to other medications. It improves sleep parameters, particularly TST and SE, with optimal efficacy observed at 5.7 mg; however, due to limited long-term safety data and children’s sensitivity, 1–2 mg is recommended [167]. Age-stratified analyses indicate that treatment response may increase with age. Beyond sleep, melatonin also benefits children with neurodevelopmental disorders, improving behavior, mood, communication, and sensory regulation, with durable effects maintained in 76% of patients [167]. These findings reinforce melatonin as the first-line pharmacological option for pediatric insomnia in ASD, although standardized dosing protocols and long-term data are still needed.

#### 6.3.2. α2-Adrenergic Agonists

α2-adrenergic agonists are approved for children with ADHD symptoms and are increasingly used off-label in individuals with ASD [125].

Clonidine, a selective α2-adrenergic receptor agonist, acts centrally and peripherally to inhibit noradrenergic transmission via presynaptic mechanisms, improving impulsivity and hyperactivity by modulating prefrontal noradrenergic tone [168,169]. In two small RCTs, clonidine showed efficacy in ASD, reducing hyperactivity, hyperarousal, stereotypies, aggressiveness, mood instability, and irritability [170,171]. In addition to behavioral benefits, clonidine has been reported to improve sleep quality by reducing SoL and WASO as demonstrated in a retrospective study, with main adverse effects including skin irritation with transdermal formulations and daytime drowsiness with oral administration [168]. Dosages are reported in Table 2.

Guanfacine, another α2-agonist approved for ADHD, acts predominantly on postsynaptic receptors in the prefrontal cortex, enhancing noradrenergic signaling and strengthening network connectivity. In ASD, it attenuates symptoms such as hyperactivity, impulsivity, and stereotypies [172,173]. However, its effects on sleep remain unclear. While some studies reported insomnia improvement in children with ASD [174], a randomized, placebo-controlled trial of extended-release guanfacine in children with ASD and ADHD did not show significant benefits on sleep [172].

The evidence supporting the use of α2-adrenergic agonists in pediatric ASD remains limited. Only a few randomized controlled trials are available [171,172,173], with small sample sizes and relatively short follow-up, while most of the other studies are open-label or retrospective [168,170]. Furthermore, outcomes have been assessed through standardized clinical scales rather than objective or neurophysiological measures, which limits the strength and generalizability of current findings [168,170,171,172,173].

#### 6.3.3. Antihistamines

First-generation antihistamines are commonly used over-the-counter agents for pediatric sleep disturbances. They are liposoluble, cross the blood–brain barrier, and act on central H1 receptors with minimal effects on sleep architecture. Table 2 summarizes the most frequently used compounds and their dosages.

Diphenhydramine, a competitive H1 receptor antagonist, is the most widely used and reaches peak plasma and tissue concentrations within 2 h of ingestion [125,175,176]. Other agents include trimeprazine, niaprazine, and hydroxyzine, the latter considered the safest [177,178]. Evidence on trimeprazine is inconsistent, ranging from no sustained benefit to reduced nocturnal awakenings and increased TST in young children with stable short-term efficacy [179,180]. Another study suggested trimeprazine may be effective when combined with behavioral strategies [181]. However, despite their widespread use, no RCT are available in pediatric populations and adverse effects are frequent, including sedation, anticholinergic symptoms, and paradoxical excitation [106,125,182,183]. Given their unfavorable tolerability profile, rapid tolerance, and risk of paradoxical reactions, antihistamines are not recommended as a first-line treatment for sleep disturbances in children with NDDs [184]. Overall, the evidence supporting the use of first-generation antihistamines in children with neurodevelopmental disorders is limited [106]. Most studies have small sample sizes, short follow-up periods, and outcomes primarily assessed through subjective reports rather than standardized or objective sleep measures [175,177,180]. Adverse effects, including sedation and other general effects, are common, and tolerance can develop rapidly [106,175,178]. Further well-designed controlled studies are needed to clarify their safety and efficacy.

#### 6.3.4. Antipsychotics

Antipsychotics act primarily through postsynaptic D2 receptor blockade, reducing mesolimbic dopaminergic hyperactivity implicated in psychosis, mania, and aggression [185,186]. They are categorized into typical (first-generation) and atypical (second-generation), which differ in their binding affinities for D2 dopamine receptors; atypical antipsychotics additionally antagonize 5-HT2A receptors. This dual mechanism accounts for their broader efficacy and lower risk of extrapyramidal symptoms and hyperprolactinemia, making them the preferred agents [187]. Although risperidone and aripiprazole are approved for ASD-related behavioral symptoms, other antipsychotics are typically used off-label to manage severe disruptive behaviors in children [188,189].

In ASD, evidence for antipsychotics in managing sleep disturbances is limited; however, atypical agents such as risperidone, aripiprazole, olanzapine, and quetiapine have been associated with improved sleep quality [39]. Quetiapine has shown concurrent reductions in aggressive behaviors and sleep improvements, as reported in a small observational study of high-functioning adolescents with ASD treated with low doses [190]. Risperidone and olanzapine have also been prescribed for sleep problems, although controlled evidence remains lacking [106,191,192]. Dosages are reported in Table 2.

However, studies on the effects of antipsychotics on sleep disturbances present several limitations, such as small sample size, lack of a control group or reliance on subjective and at times largely non-specific outcome measures for ASD, which reduce the strength and generalizability of their findings [188,189]. Furthermore, outcomes are sometimes assessed within a limited age range and without adequately accounting for potential confounding factors (such as environmental influences) that may affect behavior and, consequently, clinical outcomes during the observation period [190]. Among various studies on the use of antipsychotics in pediatric subjects with ASD, only Kent and colleagues provided valuable longitudinal data through a 26-week, flexible-dose open-label extension following a 6-week, fixed-dose, double-blind, randomized, placebo-controlled trial. However, despite the multicenter design and repeated safety and efficacy assessments, sleep evaluation was based solely on a subjective rating scale [192]. Moreover, the meta-analysis conducted by Leucht and colleagues highlighted key limitations across studies, including a limited evidence base for low- and mid-potency first-generation antipsychotics, and the predominant use of haloperidol as a comparator, which may have overestimated extrapyramidal side effects relative to atypical agents [187].

Given their adverse metabolic profile and risk of weight gain, atypical antipsychotics are not recommended as primary treatment for pediatric insomnia. Their use should be restricted to cases with severe behavioral comorbidities, where improvements in sleep may occur as a secondary effect [125].

#### 6.3.5. Benzodiazepines and Z-Drugs

Benzodiazepines and Z-drugs share a common mechanism of action through the GABA-A receptor, enhancing inhibitory neurotransmission, but with different binding profiles. Benzodiazepines bind to the benzodiazepine site, while Z-drugs act more selectively on the benzodiazepine-1 subtype, offering pharmacokinetic advantages such as shorter half-life and fewer residual daytime effects [39,125,193,194,195]. Among benzodiazepines, clonazepam has demonstrated some benefits in reducing nocturnal awakenings, nightmares, and abnormal motor behaviors during sleep, mainly in small case series of children with Williams Syndrome and isolated ASD cases [196,197]. In comparison, Z-drugs effectively reduce SoL and improve perceived sleep quality, although effects on TST remain inconsistent [194,195]. Limited pediatric data suggest Zolpidem generally shows mild, self-limiting adverse events in children [198,199]. A randomized placebo-controlled crossover trial is currently evaluating zolpidem specifically for sleep in children with ASD (NCT05540574) [200]. Zaleplon, with its rapid onset and short duration of action, may be helpful for sleep-onset difficulties; however, its efficacy and safety have been evaluated only in adult populations [201]. Dosages are summarized in Table 2.

However, both classes present important limitations. Benzodiazepines are rarely used in children due to insufficient pediatric evidence, the risk of cognitive impairment, dependence liability, and reports of paradoxical reactions such as disinhibition, hyperactivity, or irritability, largely based on clinical impressions and studies in adults with IDD [193,202,203]. Moreover, a recent meta-analysis found that ASD patients may present a reduced response to benzodiazepines, even in clinical conditions where they are usually considered highly effective first-line treatment [204]. Their use is therefore restricted to short-term or occasional indications, such as parasomnias or RLS, pending further research to clarify safety and efficacy in ASD [125,205]. Similarly, Z-drugs are not approved for individuals under 18 years, and pediatric data remain insufficient. Although their risk of tolerance and dependence appears lower than benzodiazepines, it is still present [206]. In addition, common side effects include headache, gastrointestinal symptoms, dizziness, and next-day cognitive or psychomotor impairment, while zaleplon may suppress REM sleep [194,195,201,207]. To date, there is no strong evidence from RCT supporting their safety or efficacy in children with ASD [39,208]. Only a limited number of RCTs exist, and these have been conducted primarily in adult patients with sleep disturbances [198,201].

Overall, while both benzodiazepines and Z-drugs act through GABAergic modulation and may transiently improve specific sleep parameters, their limited evidence base, safety concerns, and potential adverse effects constrain their clinical use in pediatric ASD populations.

#### 6.3.6. Antidepressants

Tricyclic and atypical antidepressants (mirtazapine, nefazodone, and trazodone) are often prescribed for pediatric insomnia due to their sedative effects and modulation of neurotransmitters involved in sleep regulation, including serotonin, histamine, and acetylcholine [125]. Evidence in children with NDDs is limited and mostly derived from small studies. Amitriptyline is used off-label in clinical practice, but its role is constrained by risks such as anxiety, agitation, anticholinergic effects, and cardiotoxicity (dosages in Table 2) [125]. Mirtazapine improves SoL and WASO with minimal impact on REM sleep [209]. An open-label study involving 26 individuals aged 3.8 to 23.5 years reported moderate benefits in children with ASD or other pervasive developmental disorders, also reducing behavioral symptoms with mild and transient side effects [210]. Trazodone, through 5-HT2 and H1 antagonism and possible modulation of melatonin pathways, is commonly used for insomnia in children with comorbid mood or anxiety disorders [211]. It may enhance sleep quality and memory in individuals with IDD, although pediatric evidence remains limited [125,212]. Across agents, common adverse effects include daytime sedation, nausea, and morning grogginess, while rarer risks include serotonin syndrome, hypotension, tachycardia, and priapism [213].

Despite their use in clinical practice, no RCTs have evaluated antidepressants for sleep disturbances in children with ASD. To date, the available evidence supports mirtazapine through a single open-label study, showing moderate benefits but with methodological limitations [210].

#### 6.3.7. Anti-Seizure Medications

Among anti-seizure medications, gabapentin, approved for neuropathic pain and RLS, has shown benefits on sleep architecture by reducing WASO and increasing NREM sleep, at doses lower than those used for seizure control [214]. In a small open-label study of 23 children, mostly with NDDs, gabapentin was reported to be safe and well-tolerated for the treatment of insomnia, improving both sleep onset and maintenance difficulties. Mild adverse effects, such as agitation and difficulties falling asleep, were occasionally observed [215]. Dosages are reported in Table 2.

#### 6.3.8. Orexin Receptor Antagonists

Orexins (A and B) are hypothalamic neuropeptides acting via orexin receptors type 1 and type 2 (OX1R and OX2R, respectively), crucial for sleep–wake regulation through promotion of arousal, suppression of sleep, and modulation of feeding behavior [125]. Dysregulated orexin signaling, particularly nocturnal hyperactivity, has been linked to insomnia and other sleep–wake disorders [216]. Based on this, orexin receptor antagonists (ORAs), either selective (SORAs) or dual (DORAs), have emerged as a promising therapeutic strategy. DORAs such as suvorexant, lemborexant, and daridorexant promote sleep by blocking orexin receptors, thereby dampening arousal and facilitating sleep onset and maintenance [216]. Experimental data further suggest potential cognitive advantages: in murine hippocampal slices, OX2R activation shortened ripple duration, while DORAs did not alter sharp-wave duration, indicating that they may reduce orexinergic activity without impairing learning and memory [217]. These findings support the potential of DORAs as safer options for long-term use. Importantly, ORAs also present practical advantages, including a unique mechanism of action and minimal pharmacodynamic interaction with medications commonly prescribed in ASD, which makes them particularly appealing in this population. However, evidence in pediatric cohorts is still lacking. Robust RCTs are needed to establish short- and long-term efficacy and safety, especially in children with ASD and comorbid sleep disturbances [125].

## 7. Future Directions

Effective management of sleep disorders in ASD requires accurate diagnosis and recognition of overlapping neurological systems in comorbid conditions. The first step should always include sleep hygiene and behavioral strategies, which are essential to achieve sustained long-term improvements and to provide families with structured coping resources.

**Figure 2:**
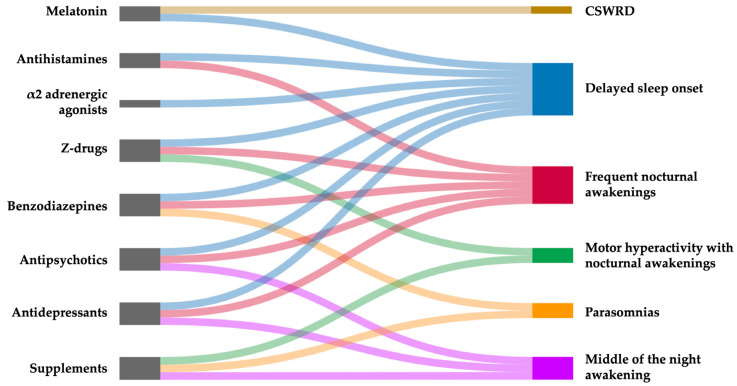
Sankey diagram depicting the relationships between pharmacological classes (left) and specific sleep disturbances (right), including delayed sleep onset, frequent nocturnal awakenings, motor hyperactivity with nocturnal awakenings, parasomnias, middle-of-the-night awakening, and circadian sleep–wake rhythm disorder (CSWRD). While each link represents a single therapeutic association, the relative width of the nodes reflects the number of connections, indicating on the left the range of sleep problems addressed by each medication class, and on the right the variety of pharmacological approaches available for each disorder.

When these measures are insufficient, pharmacological treatments may be introduced. Figure 2 summarizes the role of various pharmacological options for managing sleep disorders in individuals with ASD.

Melatonin remains the first-line treatment due to its favorable safety profile and physiological basis. Other agents, such as clonidine in selected comorbidities, or mirtazapine when sleep difficulties co-occur with mood symptoms, may be considered. Antipsychotics may be used in case of comorbid indications. While preliminary evidence on ORAs is promising, RCTs in pediatric ASD populations are urgently needed, especially to clarify long-term safety and tolerability. In addition, supplementation with promising agents such as Trp, as well as iron and magnesium in conditions like RLS and PLMD, should be considered as part of an individualized treatment approach. Despite the widespread clinical use of these nutritional supplements, large-scale randomized controlled trials are lacking to establishing their efficacy, safety, and optimal dosing in children with ASD. This gap is especially noticeable for vitamin D, whose potential role in sleep regulation and neurodevelopment has yet to be systematically investigated in pediatric ASD populations.

Beyond pharmacological interventions, technological advances open new avenues for sleep management. Wearable EEG devices and actigraphy, combined with AI-assisted analytics, could provide objective biomarkers of sleep architecture and circadian rhythms, enabling earlier identification of sleep disturbances and stratification of patients [218,219,220]. Likewise, digital behavioral interventions, such as app-based parent training programs, represent scalable strategies to increase accessibility, ensure treatment fidelity, and provide ongoing support to families [221]. Integrating these innovations with individualized clinical care is valuable to move from fragmented approaches toward personalized, multimodal treatment strategies that combine sensory, behavioral, and medical interventions, while also supporting longitudinal research on the long-term effects of early sleep interventions on neurodevelopment.

## 8. Conclusions

Sleep disturbances in ASD represent both a pervasive clinical challenge and a unique therapeutic window. A growing body of evidence underscores their bidirectional relationship: disrupted sleep during sensitive periods of brain development may interfere with synaptic pruning, sensory integration, and circuit maturation, thereby amplifying atypical neurodevelopment. Conversely, core features of ASD—such as sensory hypersensitivity and excitatory/inhibitory imbalance—can further impair sleep regulation, creating a self-reinforcing cycle.

Therefore, effective clinical management of sleep disturbances mitigates nocturnal symptoms, promotes neuroplasticity and neural circuit maturation, enhances daytime cognitive and adaptive functioning, and improves family well-being. Progress in this field requires not only rigorous evaluation of novel pharmacological agents (e.g., ORAs) but also the integration of wearable EEG, actigraphy, and AI-assisted monitoring to identify reliable biomarkers and refine treatment decisions. Equally, digital behavioral interventions, including app-based parent training, offer scalable solutions to extend access and sustainability of care.

Ultimately, the management of sleep disturbances in ASD should be personalized to the child’s developmental profile, clinical needs, and comorbidities, embedding pharmacological, behavioral, and digital tools within precision medicine frameworks. Prioritizing sleep interventions is a key neuroprotective strategy that supports developmental trajectories, improving daily functioning and quality of life for affected children and their families. In this perspective, sleep should be considered not only a symptom to be managed but a modifiable determinant of developmental outcomes in ASD.

## Figures and Tables

**Table 1:** Behavioral interventions for sleep disturbances in children with ASD.

Behavioral Intervention	Description of the Intervention	Type of Sleep Disorder
Extinction—planned ignoring	Caregiver ignores undesirable, sleep-disruptive behavior (e.g., crying), encouraging self-soothing.	Delayed sleep onset, nocturnal awakenings, co-sleeping, and bedtime resistance
Gradual extinction	Caregiver ignores bedtime disruption only for a predetermined amount of time before intervening.	
Scheduled awakenings	Caregiver interrupts the sleep cycle and prevents the disruptive episode by waking the child shortly before the typical onset of the event.	Arousal disorders such as nocturnal awakenings and sleep terrors
Bedtime fading	Gradually delay bedtime to match the child’s natural sleep-onset time.	Bedtime resistance
Stimulus fading	Caregiver progressively increases their physical distance from the child at bedtime.	Co-sleeping
Chronotherapy	Caregiver gradually moves bedtime and wake time later each day to shift and stabilize the child’s circadian rhythm.	CSWRD
Bedtime pass	The child receives a bedtime pass allowing one room exit or parent visit per night. After use, further exits are ignored, with a silent return to bed. Unused passes can be traded for a weekly reward.	Nocturnal awakenings

Legend: ASD: Autism Spectrum Disorder; CSWRD: circadian sleep–wake rhythm disorder

**Table 2:** Sleep-Promoting Medications: Mechanisms, Effects on Sleep Architecture, and Safety.

Agents	Pharmacological Class	Dosage	Mechanisms of Action	Effect on Sleep Structure	Adverse Effects
Melatonin	Serotonin-derived neurohormone	0.5–4 mg/day up to 6 mg in adolescents	MT1/MT2 R agonist, contributing to sleep-promoting and chronobiotic effects	↓ SoL, ↑ TST, regulate circadian rhythms	Daytime drowsiness, headache, nausea
Clonidine	α2-adrenergic agonist	0.05–0.10 mg/day up to 0.30 mg/day	α2-adrenergic R agonist, inhibiting noradrenergic transmission	↑ REM latency	Daytime drowsiness, orthostatic hypotension, GI disorder, skin irritation (due to transdermal formulations)
Diphenhydramine	Antihistamine	0.5 mg/kg up to 25 mg/day	H1 R antagonist	↓ SoL, ↓ arousal threshold	Daytime drowsiness, GI disorder; overdose leading to catatonic stupor, anxiety, hallucinations, respiratory failure
Trimeprazine tartrate	Antihistamine	6 mg/kg/day	H1 R and D2 R antagonist	↓ WASO and NA	Daytime drowsiness, irritability
Hydroxyzine	Antihistamine	0.5–1 mg/kg/day	H1 R antagonist	↓ SoL, ↓ arousal threshold	Daytime drowsiness, headache
Niaprazine	Antihistamine	1 mg/kg/day	H1 R antagonist	↓ SoL, ↓ arousal threshold	Daytime drowsiness
Clonazepam	Benzodiazepine	0.25–0.5 mg/day	GABA Rs agonist	↓ SoL, ↓ arousal threshold	Daytime drowsiness, rebound insomnia, anterograde amnesia (dose-dependent); respiratory depression
Zolpidem	Z-drug	0.25 mg/kg/day up to 10 mg/day	GABA Rs agonist	↓ SoL and WASO, ↑ TST	Headache, GI disorders, dizziness
Mirtazapine	Antidepressant	7.5–45 mg/day	Presynaptic α2-adrenergic R and 5-HT2/3 R antagonist	↑ SWS and ↓ SoL	Sedation, dry mouth, ↑ appetite, GI disorders, myalgia, dizziness, tremor, irritability, confusion
Trazodone	Antidepressant	25–150 mg/day	5-HT2A/C R and H1 R antagonist	↓ SoL, ↑ sleep maintenance, ↓ REM, ↑ SWS	Daytime drowsiness, nausea, morning grogginess, serotonin syndrome, hypotension
Amitriptyline	Antidepressant	5–50 mg/day	5-HT2A R, α-adrenergic, H1 R, and mACh R antagonist	↓ SoL, ↑ REM latency	Anxiety, constipation, sedation, anticholinergic effects, cardiotoxicity
Gabapentin	Anti-seizure medication	3–15 mg/kg/day	GABA Rs agonist	↑ SWS and ↓ WASO	Irritability and delayed sleep onset
Risperidone	Atypical antipsychotic	0.5–3.5 mg/day	D2 R, and 5-HT2 R antagonist	↓ SoL and WASO, ↑ TST and SWS	Weight gain, DM, hyperlipidemia, neuroleptic malignant syndrome, tardive dyskinesia, MD
Aripiprazole	Atypical antipsychotic	1–5 mg/day	D2 R antagonist or partial agonist; 5-HT1A/2C R partial agonist, 5HT2A R antagonist	↓ SoL and WASO, ↑ TST and SWS	Weight gain, DM, hyperlipidemia, neuroleptic malignant syndrome, tardive dyskinesia, MD
Olanzapine	Atypical antipsychotic	2–10 mg/day	D2/D4 R antagonist and 5-HT2A/2C R antagonist	↓ SoL and WASO, ↑ TST and SWS	Weight gain, daytime drowsiness, hypercholesterolemia, DM
Quetiapine	Atypical antipsychotic	25–150 mg/day	D2 R, 5-HT1A/2A R antagonist	↓ SoL and WASO, ↑ TST and SWS	Weight gain, dizziness, headache, sedation, orthostatic hypotension, hyperglycemia, dyslipidemia, tardive dyskinesia
Suvorexant	Orexin antagonist	10 mg/day up to 20 mg	OX1/2 R antagonist	↓ SoL and WASO	Daytime drowsiness, headache, abnormal dreams, narcolepsy/cataplexy

Legend: ↑: increase; ↓: decrease; 5-HT1A/2A/2C/3 R: serotonin receptor type 1A/2A/2C/3; DM: diabetes mellitus; D2/D4 R: dopamine receptor type 2/type 4; GABA: **γ**-aminobutyric acid; GI: gastrointestinal; H1 R: Histamine receptor type 1; mACh R: muscarinic acetylcholine receptors; MD: movement disorders; mg: milligram; MT1/MT2 R: Melatonin receptor type 1/type 2; NA: nocturnal awakenings; OX1/2: orexin receptor type1/type2; REM: rapid eye movements; SoL: Sleep onset latency; SWS: slow-wave sleep; TST: total sleep time; WASO: Wake after sleep onset.

## Abbreviations

The following abbreviations are used in this manuscript:

5-HTP	5-Hydroxitryptophan
ADHD	Attention Deficit Hyperactivity Disorder
ASD	Autism Spectrum Disorder
CSWRD	Circadian Sleep–Wake Rhythm Disorder
CSHQ	Children’s Sleep Habits Questionnaire
DLMO	Dim Light Melatonin Onset
DORAs	Dual Orexin Receptor Antagonists
EEG	Electroencephalographic
EMG	Electromyogram
FDA	U.S. Food and Drug Administration
FR	Fast Release
GABA	Gamma-aminobutyric acid
IDD	Intellectual Developmental Disorder
IQ	Intelligence Quotient
MT	Melatonin receptor
NDDs	Neurodevelopmental Disorders
NREM	Non-Rapid Eye Movement
ORAs	Orexin Receptor Antagonists
OXR	Orexin Receptor
PedPRM	Pediatric Prolonged Release Melatonin
PLMD	Periodic Limb Movement Disorder
RCTs	Randomized Controlled Trials
REM	Rapid Eye Movement
RLS	Restless Legs Syndrome
RSD	Restless Sleep Disorder
SE	Sleep Efficiency
SoL	Sleep onset Latency
SORAs	Selective Orexin Receptor Antagonists
SSP	Short Sensory Profile
SSRIs	Selective Serotonin Reuptake Inhibitors
SWA	Slow-Wave Activity
SWR	Sharp Wave Ripple
Trp	Tryptophan
TST	Total Sleep Time
UVB	Ultraviolet B
WASO	Wake After Sleep Onset
